# Association between Arsenic Exposure from Drinking Water and Longitudinal Change in Blood Pressure among HEALS Cohort Participants

**DOI:** 10.1289/ehp.1409004

**Published:** 2015-03-27

**Authors:** Jieying Jiang, Mengling Liu, Faruque Parvez, Binhuan Wang, Fen Wu, Mahbub Eunus, Sripal Bangalore, Jonathan D. Newman, Alauddin Ahmed, Tariqul Islam, Muhammad Rakibuz-Zaman, Rabiul Hasan, Golam Sarwar, Diane Levy, Vesna Slavkovich, Maria Argos, Molly Scannell Bryan, Shohreh F. Farzan, Richard B. Hayes, Joseph H. Graziano, Habibul Ahsan, Yu Chen

**Affiliations:** 1 Department of Population Health, New York University School of Medicine, New York, New York, USA; 2 Department of Environmental Health Sciences, Mailman School of Public Health, Columbia University, New York, New York, USA; 3 U-Chicago Research Bangladesh Ltd., Dhaka, Bangladesh; 4 The Leon H. Charney Division of Cardiology, Department of Medicine, New York University School of Medicine, New York, New York, USA; 5 Department of Biostatistics, Mailman School of Public Health, Columbia University, New York, New York, USA; 6 Department of Health Studies, The University of Chicago, Chicago, Illinois, USA; 7 Department of Community and Family Medicine, Geisel School of Medicine at Dartmouth, Lebanon, New Hampshire, USA; 8 Department of Medicine,; 9 Department of Human Genetics, and; 10 Comprehensive Cancer Center, The University of Chicago, Chicago, Illinois, USA

## Abstract

**Background:**

Cross-sectional studies have shown associations between arsenic exposure and prevalence of high blood pressure; however, studies examining the relationship of arsenic exposure with longitudinal changes in blood pressure are lacking.

**Method:**

We evaluated associations of arsenic exposure in relation to longitudinal change in blood pressure in 10,853 participants in the Health Effects of Arsenic Longitudinal Study (HEALS). Arsenic was measured in well water and in urine samples at baseline and in urine samples every 2 years after baseline. Mixed-effect models were used to estimate the association of baseline well and urinary creatinine-adjusted arsenic with annual change in blood pressure during follow-up (median, 6.7 years).

**Result:**

In the HEALS population, the median water arsenic concentration at baseline was 62 μg/L. Individuals in the highest quartile of baseline water arsenic or urinary creatinine-adjusted arsenic had a greater annual increase in systolic blood pressure compared with those in the reference group (β = 0.48 mmHg/year; 95% CI: 0.35, 0.61, and β = 0.43 mmHg/year; 95% CI: 0.29, 0.56 for water arsenic and urinary creatinine-adjusted arsenic, respectively) in fully adjusted models. Likewise, individuals in the highest quartile of baseline arsenic exposure had a greater annual increase in diastolic blood pressure for water arsenic and urinary creatinine-adjusted arsenic, (β = 0.39 mmHg/year; 95% CI: 0.30, 0.49, and β = 0.45 mmHg/year; 95% CI: 0.36, 0.55, respectively) compared with those in the lowest quartile.

**Conclusion:**

Our findings suggest that long-term arsenic exposure may accelerate age-related increases in blood pressure. These findings may help explain associations between arsenic exposure and cardiovascular disease.

**Citation:**

Jiang J, Liu M, Parvez F, Wang B, Wu F, Eunus M, Bangalore S, Newman JD, Ahmed A, Islam T, Rakibuz-Zaman M, Hasan R, Sarwar G, Levy D, Slavkovich V, Argos M, Scannell Bryan M, Farzan SF, Hayes RB, Graziano JH, Ahsan H, Chen Y. 2015. Association between arsenic exposure from drinking water and longitudinal change in blood pressure among HEALS cohort participants. Environ Health Perspect 123:806–812; http://dx.doi.org/10.1289/ehp.1409004

## Introduction

There is a strong and direct relationship between high blood pressure (BP) and cardiovascular disease (CVD) mortality (Lewington et al. 2002). High BP remains prevalent in the United States and internationally among adults over the age of 35 years (Chobanian et al. 2003; Frohlich 1997). Rapid increases in the prevalence of high BP in low-income countries (Gupta and Gupta 2009; Ibrahim and Damasceno 2012; Lawes et al. 2003; Redon et al. 2011) has likely contributed to the rising epidemic of CVD in these populations (Ibrahim and Damasceno 2012). In recent decades, there has been growing awareness of the potential importance of environmental factors such as mercury (Houston 2011), lead (Navas-Acien et al. 2007), cadmium (Eum et al. 2008), and arsenic (Abhyankar et al. 2012) in the development of high BP. The identification and mitigation of environmental exposures associated with high BP may help reduce CVD risk (Abhyankar et al. 2012).

Previous studies have indicated associations between exposure to inorganic arsenic and the development of vascular diseases, including high BP, peripheral vascular diseases, and ischemic heart disease (Abhyankar et al. 2012; Chen CJ et al. 1996; Chen Y et al. 2007a; Tseng et al. 1996). A systematic review examining 11 cross-sectional studies on arsenic exposure and the prevalence of high BP (Abhyankar et al. 2012) found that 10 of the 11 studies reported a positive association, whereas only one study indicated no association. The review included 8 studies with arsenic levels of moderate to high (average ≥ 50 μg/L), and 3 studies with relatively low arsenic levels (average < 50 μg/L). However, prospective cohort studies that can better characterize the association between arsenic and high BP are lacking. Longitudinal studies with repeated measurements of BP, which provide a powerful tool to evaluate health outcomes that change over time, are needed to assess whether arsenic is associated with increasing BP over time.

It is estimated that millions of Americans are exposed to drinking water with arsenic concentrations exceeding the World Health Organization (WHO) standard (10 μg/L) (U.S. Environmental Protection Agency 2000). In Bangladesh, where the majority of the population relies on groundwater and arsenic contamination of wells is widespread, > 50 million people have been chronically exposed (British Geological Survey 2007). In 2000, we established the Health Effects of Arsenic Longitudinal Study (HEALS), a large prospective cohort study of 11,746 individuals in Araihazar, Bangladesh, to assess the health effects of arsenic exposure. In cross-sectional analyses using participants’ baseline data, we previously reported a positive association between baseline arsenic exposure, measured either in urine or drinking water samples, and BP (Chen Y et al. 2007a). To characterize the rate of BP changes related to arsenic exposure, we assessed the association of baseline arsenic exposure (measured both in water and urine) with longitudinal changes in BP among 10,853 participants in Bangladesh who had well water arsenic concentrations ranging from 0.1 to 864 μg/L (median = 62 μg/L).

## Materials and Methods

*Study population.* HEALS is an ongoing prospective cohort study in Araihazar, Bangladesh. The principle aim of HEALS is to investigate the health effects of arsenic in drinking water. A detailed description of the cohort has been presented elsewhere (Ahsan et al. 2006). Briefly, before recruitment, water samples were collected for a set of 5,966 continuous wells in a well-defined geographic area of 25 km^2^ in Araihazar. Between October 2000 and May 2002, 11,746 men and women 18–75 years of age were recruited who met the following criteria: *a*) married male or female (to reduce loss to follow-up), *b*) resident of the study area for at least 5 years, and *c*) primarily used drinking water from 1 of the 5,966 study wells for at least 3 years (Ahsan et al. 2006), leading to a response rate of 97.5% (original cohort). HEALS was expanded to include an additional 8,287 participants in 2007–2008 (expansion cohort) following the same methodologies (Wu et al. 2011). The present study focused on the original cohort because these individuals were followed for a longer period of time (median, 6.7 years; range, 0.9–8.3 years). Baseline interviews were conducted to gather information regarding history of well water use, demographics, and lifestyle characteristics. The cohort is being actively followed, with follow-up assessments conducted roughly every 2 years. The current analysis included data from the first (September 2002–May 2004), second (September 2004–May 2006), and third (June 2007–March 2009) follow-ups, at which time a physical examination, collection of urine samples, and a structured interview were conducted using the same procedures as those used in the baseline interview. Informed consent was obtained from study participants, and study procedures were approved by the ethics committee of the Bangladesh Medical Research Council and the institutional review boards of Columbia University and the University of Chicago.

For the present study, we excluded individuals who died before the first follow-up (*n* = 107), those taking hypertension treatment at baseline (*n* = 126), those without systolic BP (SBP) or diastolic BP (DBP) measurements at baseline (*n* = 380), and individuals for whom no measurements of SBP or DBP were recorded during the follow-up (*n* = 406). The final study population was 10,853. The distributions of demographic and lifestyle factors between the overall population and the study population were very similar (see Supplemental Material, Table S1).

*Measurements of arsenic exposure.* In rural Bangladesh, the majority of the population uses a single hand-pumped tube well for their primary source of drinking water. There is no municipal water treatment. Water samples from 5,966 tube wells were collected in 50-mL acid-washed bottles after pumping each well for 5 min. Samples were immediately acidified using 1% HCl until December 2003, after which samples were acidified at Columbia University, normally several months after collection, because delayed acidification does not affect measurement results (van Geen et al. 2007). Total arsenic concentration was first determined by graphite furnace atomic absorption spectrometry (GFAA), with a detection limit of 5 μg/L. If water samples were found to have arsenic concentrations at or below the detection limit of GFAA, they were then analyzed by high-resolution inductively coupled plasma mass spectrometry (HR ICPMS), with a detection limit of < 0.1 μg/L (Chen Y et al. 2007b). The long-term reproducibility determined from consistency standards included with each run is relatively stable over time (Cheng et al. 2004, 2005; van Geen et al. 2005).

Spot urine samples were collected in 50-mL acid-washed tubes from 95.6, 94.5, 91.6, and 89.9% of the cohort participants at baseline and at the first, second, and third follow-up visits, respectively. Total arsenic concentration was measured by GFAA spectrometry using a PerkinElmer (Waltham, MA) AAnalyst 600 graphite furnace system with a detection limit of 2 μg/L, as previously described (Nixon et al. 1991). Urinary creatinine was analyzed using a method based on the Jaffe reaction for adjustment of urinary total arsenic concentration (Slot 1965). The median of creatinine concentration at baseline was 52.3 mg/dL (range, 2.8–376.0) for men and 41.5 mg/dL (range, 1.3–303.1) for women.

Given that drinking water was the main source of arsenic exposure in the population (see Supplemental Material, “Details on arsenic exposure in the population”) and urinary creatinine-adjusted arsenic can reflect internal dose of exposure (Marchiset-Ferlay et al. 2012), we used both as indicators for arsenic exposure. At baseline, in order to help improve the health of the community and reduce their risks from arsenic exposure, an arsenic mitigation program was implemented to promote switching to wells with relatively lower water arsenic concentration (< 50 μg/L) (Chen Y et al. 2007b). At the first follow-up, a total of 58% of the 6,512 participants who consumed well water with arsenic concentrations ≥ 50 μg/L at baseline had switched to nearby wells. However, among those individuals that switched wells, only 27% participants had switched to wells with lower arsenic concentrations (i.e., < 50 μg/L) (Chen Y et al. 2007b). We used urinary creatinine-adjusted arsenic assessed at follow-up visits to track the change in exposure during follow-ups (Marchiset-Ferlay et al. 2012). Because arsenic level remained similar in the majority of the participants (see Supplemental Material, “Details of arsenic exposure in the population”), the impact of visit-to-visit change of urinary creatinine-adjusted arsenic on BP change was considered short-term compared with urinary creatinine-adjusted arsenic at baseline, which reflected exposure from the baseline wells that participants had used for an average of 8.6 (median, 7; range, 3–50) years prior to baseline (Chen Y et al. 2010, 2011, 2013a, 2013b), and thus visit-to-visit change of urinary creatinine-adjusted arsenic was not considered as the main exposure of interest.

*BP measurements.* BP was measured at baseline and at each follow-up by trained clinicians using an automatic sphygmomanometer (HEM 712-C; Omron Healthcare GmbH, Hamburg, Germany), which has been validated to have 85% of readings falling within 10 mmHg (O’Brien et al. 2001). Measurements were taken with participants in a seated position after 5 min of rest, with the cuff around the upper left arm, in accordance with recommended guidelines (Pickering et al. 2005). Two BP measurements were taken at follow-ups, and we used the arithmetic mean of two for the analyses. The reliability of the BP measurement was high, with all intraclass correlation coefficients between 0.92 and 0.94 at a given visit (Chen Y et al. 2007a).

The participation rate for the first, second, and third follow-ups, respectively, were 96.9, 93.6, and 92.2% of the cohort participants at baseline. Information on medication use was collected at baseline and during follow-ups. Study participants were asked about all medicines they were taking regularly, and were asked to show the medications or prescriptions to the interviewers. Medications were standardized to generic names and then sorted into one of 44 medication categories (Scannell et al. 2013). Participants who reported taking antihypertensive medication were identified for the present study.

*Lifestyle characteristics.* Lifestyle characteristics were measured at baseline and follow-ups, or only at baseline. Past or current use of cigarette smoking was ascertained in the questionnaire at each follow-up. Diabetes status was identified by asking participants if they were diagnosed with diabetes by a physician. Previously reported comparisons between self-reported diabetes status in our study and test results for glycosylated hemoglobin and glucosuria showed that only 1% of the individuals without self-reported diabetes tested positive on urinary glucose, whereas 61% of the individuals with self-reported diabetes tested positive (*p* < 0.01), which indicated good questionnaire validity (Chen Y et al. 2010, 2011). Body mass index (BMI) was calculated based on measured height and weight (kilograms per meter squared). Educational status was obtained at baseline at each follow-up.

*Statistical analysis.* We first conducted descriptive analyses to compare the distribution of demographic and lifestyle characteristics and BP measurements over time by baseline water arsenic categorized into quartiles in the overall study population.

We used longitudinal mixed-effect models with a random slope and an intercept for each subject, to assess the association between baseline arsenic, using either water or urinary creatinine-adjusted arsenic, and annual change in BP over time. The constructed mixed-effects model is a two-level model, in which the first level describes how BP changes in the population (fixed effect), while the second level of the model depicts how individual BP changes over time (random effect). The mixed-effect model also accounts for within-subject correlation between baseline and follow-up BP measurements.

We first used the mixed-effect model to assess the association of baseline demographic and lifestyle variables with annual BP change. The variables included baseline age (treated as continuous or tertile variables), sex (male, female), smoking status (never, past, current), history of diabetes (yes, no), baseline educational attainment in years (continuous or tertile variables), and BMI (kg/m^2^).

In order to investigate whether there was a dose–response relationship between long-term exposure to arsenic (either baseline water arsenic or baseline urinary creatinine-adjusted arsenic) and longitudinal BP change, arsenic concentrations were categorized into quartiles, and the mixed-effect model was also conducted as follows:

BP*_ij_* = [β_0_ + β_1_ (TIME)*_ij_* + β_2_ As_0_*_j_*_2_ + β_3_ As_0_*_j_*_3_ + β_4_ As_0_*_j_*_4_ + β_12_ As_0_*_j_*_2_ (TIME)*_ij_* + β_13_ As_0_*_j_*_3_ (TIME)*_ij_* + β_14_ As_0_*_j_*_4_ (TIME)*_ij_* + α^T^Z_0_*_j_*] + [μ_0_*_j_* + μ_1_*_j_* (TIME)*_ij_*] + *r_ij_*, [1]

where baseline arsenic exposure (either water arsenic or urinary creatinine-adjusted arsenic) was categorized into quartiles and treated with a dummy variable (As_0_*_j_*_2_, As_0_*_j_*_3_, As_0_*_j_*_4_). BP*_ij_* represents blood pressure at time *i* for subject *j*. TIME is years since baseline at the time of BP measurement; β*_k_*, *k* = 2,3,4 is the difference in mean baseline BP for baseline arsenic in the *k*th quartile compared with that in the first quartile (reference); β_1_*_k_*, *k* = 2,3,4 is the difference in annual BP change over time for baseline arsenic in the *k*th quartile compared with the reference (i.e., the estimated effect of baseline arsenic levels on annual BP change); α^T^ is a row vector of regression coefficient estimates for covariates at baseline (T denotes vector transpose); and Z_0_*_j_* is a vector of potential confounders. The random intercept μ0*_j_* and slope μ_1_*_j_* estimate the within-subject correlation among repeated measurements and between-subject heterogeneity, and *r_ij_* is the error that cannot be accounted for by other covariates and random effects. The terms in the first and second brackets, respectively, are the fixed and random parts of the model. An unstructured variance structure was specified that assumes that there was no specific pattern in the covariance matrix. BP was normally distributed at baseline and follow-ups, and was therefore not transformed. To assess the association between baseline water arsenic and annual BP change, we first adjusted for sex and age (years) (model 1). We then additionally adjusted for BMI (time dependent), smoking status (time dependent, categorized into current or not current), history of diabetes (time dependent), and educational attainment (model 2) because these variables were considered important risk factors for high BP in our population (Chen Y et al. 2007a). Because arsenic exposures may have changed from baseline levels in some participants, in the final model (model 3) we further adjusted for change in urinary creatinine-adjusted arsenic since baseline for each visit, calculated as the arsenic concentration at each follow-up minus arsenic concentration in the baseline. Similar models were constructed using baseline urinary creatinine-adjusted arsenic as the exposure variable. We also examined differences in rate of BP change associated with visit-to-visit changes in urinary creatinine-adjusted arsenic.

In all analyses, BP measurements were treated as missing for the visit when the use of antihypertension treatment was reported and thereafter. There were 126 participants being treated with antihypertension medication at baseline, 285 at the first follow-up, 412 at the second follow-up, and 658 at the third follow-up. We also conducted the same analyses using different categories of arsenic exposure (tertiles or quintiles). We conducted sensitivity analyses excluding all subjects who were ever under treatment at baseline or at any follow-up visits. We used the same equipment and protocol to measure BP at baseline and at every follow-up visit. However, because BP measurements in the second follow-up appeared to be elevated compared with BP measurements at other time points, we did a sensitivity analysis to exclude BP measurements in that follow-up.

Finally, we examined whether subjects with higher baseline arsenic exposure (water arsenic or urinary creatinine-adjusted arsenic) had higher BP at the end of follow-up. Linear regression models were used, with the arsenic exposure variables treated as categorical variables, adjusting for the same covariates. Adjusted mean levels of BP by quartiles of arsenic exposure variables were estimated using LSMEANS statement in SAS. All statistical analyses were conducted using SAS, version 9.3 (SAS Institute Inc., Cary, NC, USA). All tests conducted were two-sided, and *p* < 0.05 was considered significant.

## Results

The final study population included 10,853 participants, with median follow-up time of 6.7 years, ranging from 0.86 to 8.26 years. The median concentration was 62 μg/L for water arsenic and 88 μg/L for urinary arsenic, ranging from 0.1 to 864 μg/L and 1 to 2,273 μg/L, respectively. Of the study population, 9,070 had all four SBP measurements and 9,062 had all four DBP measurements; 1,150 had three SBP measurements and 1,159 had three DBP measurements; and 633 had two SBP measurements and 632 had two DBP measurements. There were 10,853 subjects with available water arsenic concentrations and 10,549 subjects with available baseline urinary creatinine-adjusted arsenic concentrations for analysis.

Individuals with lower baseline arsenic exposure were slightly more likely to have higher educational attainment or higher baseline BMI (Table 1). There was no significant difference in SBP or DBP by water arsenic tertile groups at baseline, first follow-up, or second follow-up. However, there were global differences in SBP and DBP measured at the third visit in relation to baseline water arsenic levels. Baseline water arsenic levels were positively associated with urinary creatinine-adjusted arsenic levels at baseline, first follow-up, second follow-up, and third follow-up.

**Table 1:** Baseline and follow-up characteristics of HEALS participants (*N *= 10,853).

Characteristic	Q1 (≤ 12 μg/L)		Q2 (12–62 μg/L)		Q3 (62–148 μg/L)		Q4 (> 148 μg/L)		*p*-Value^*a*^
	No.	Mean ± SD or %	No.	Mean ± SD or %	No.	Mean ± SD or %	No.	Mean ± SD or %	
Age (years)	2,752	36.9 ± 10.0	2,711	36.6 ± 10.0	2,688	36.6 ± 9.9	2,702	37.0 ± 10.0	0.234
Male (%)	1,170	42.5	1,143	42.2	1,124	41.8	1,151	42.6	0.935
Current smoker (%)	813	29.6	792	29.2	747	27.8	751	27.8	0.334
Diabetes history (%)	55	2.0	59	2.2	46	1.7	43	1.6	0.370
Education (years)	2,750	3.6 ± 3.9	2,710	3.2 ± 3.7	2,687	3.5 ± 3.9	2,700	3.3 ± 3.7	0.002
BMI baseline (kg/m^2^)	2,730	19.9 ± 3.3	2,701	19.7 ± 3.1	2,670	19.7 ± 3.0	2,690	19.4 ± 3.0	< 0.001
Systolic blood pressure (mmHg)
Baseline	2,752	113.6 ± 16.6	2,711	114.7 ± 17.2	2,688	113.6 ± 16.5	2,702	113.5 ± 16.6	0.391
Follow up 1	2,676	113.9 ± 16.8	2,639	113.7 ± 17.0	2,623	113.5 ± 17.1	2,639	114.2 ± 17.4	0.442
Follow up 2	2,557	118.4 ± 15.6	2,475	117.8 ± 15.3	2,492	117.8 ± 15.5	2,489	117.4 ± 15.2	0.175
Follow up 3	2,432	108.5 ± 14.9	2,399	112.6 ± 14.8	2,366	112.6 ± 15.7	2,356	112.0 ± 15.6	< 0.001
Diastolic blood pressure (mmHg)
Baseline	2,750	73.8 ± 11.5	2,711	73.6 ± 11.3	2,685	73.4 ± 11.1	2,700	73.1 ± 11.5	0.132
Follow up 1	2,677	73.0 ± 10.2	2,638	72.8 ± 10.3	2,623	72.6 ± 10.2	2,639	72.9 ± 10.3	0.557
Follow up 2	2,557	76.3 ± 10.3	2,475	76.2 ± 10.0	2,492	76.1 ± 10.0	2,489	76.0 ± 10.1	0.683
Follow up 3	2,432	71.1 ± 9.9	2,399	73.9 ± 10.0	2,366	73.8 ± 10.2	2,356	73.4 ± 10.1	< 0.001
Urinary arsenic (μg/L)
Baseline	2,711	51.0 ± 47.7	2,662	99.2 ± 79.3	2,576	150.9 ± 120.4	2,600	258.2 ± 233.8	< 0.001
Follow up 1	2,671	54.3 ± 57.7	2,633	106.9 ± 87.5	2,625	145.3 ± 133.8	2,629	185.7 ± 199.1	< 0.001
Follow up 2	2,621	54.7 ± 55.6	2,534	105.7 ± 85.5	2,547	139.1 ± 125.6	2,542	178.5 ± 191.4	< 0.001
Follow up 3	2,564	51.6 ± 57.5	2,510	92.8 ± 77.7	2,499	118.6 ± 108.8	2,484	149.2 ± 169.5	< 0.001
Urinary creatinine-adjusted arsenic (μg/g creatinine)
Baseline	2,711	99.8 ± 83.5	2,662	209.1 ± 151.7	2,576	316.8 ± 200.5	2,600	525.8 ± 488.1	< 0.001
Follow up 1	2,671	96.7 ± 74.4	2,633	193.1 ± 121.8	2,625	264.2 ± 213.6	2,629	344.9 ± 326.7	< 0.001
Follow up 2	2,621	98.5 ± 79.4	2,534	198.0 ± 120.6	2,547	259.2 ± 201.1	2,542	333.1 ± 323.1	< 0.001
Follow up 3	2,564	99.0 ± 89.5	2,510	190.2 ± 153.6	2,499	247.8 ± 191.2	2,484	305.1 ± 309.7	< 0.001
Abbreviations: Q1, quartile 1; Q2, quartile 2; Q3, quartile 3; Q4, quartile 4. Q1: median = 2.3, SD = 3.3, range = 11.9; Q2: median = 34.0, SD = 14.4, range = 49.7; Q3: median = 101.0, SD = 25.2, range = 86.0; Q4: median = 239.0, SD = 107.4, range = 714.0.^***a***^Represents the global difference and is based on the chi-square test for categorical variables and analysis of variance for continuous variables.									

The rate of annual SBP increase tended to be greater with increasing baseline age (Table 2). Age was inversely associated with the rate of longitudinal DBP increase. There was a monotonic decrease with increasing age, comparing older age groups (30–40, > 40 years of age) with younger age group (≤ 30 years of age), and the difference between the rate of DBP decrease among those > 40 years at baseline was close to being significantly lower than the rate among those ≤ 30 years at baseline. The data are consistent with previous literature that documented a decreasing DBP with increasing age (Wright et al. 2011). The annual increase in SBP was greater in women compared with men, in those with higher educational attainment than subjects with a lower educational attainment, and in those with a baseline BMI > 20.45 kg/m^2^ compared with 18.09–20.45 kg/m^2^.

**Table 2:** Relation of baseline characteristics and adjusted annual changes in blood pressure over 7 years of follow-up.*^a^*

Baseline characteristic	SBP change/year (mmHg) β (95% CI)	*p*-Value	DBP change/year (mmHg) β (95% CI)	*p*-Value
Age (years)^*b*^
≤ 30	Reference		Reference
30–40	0.14 (0.02, 0.27)	0.020	–0.05 (–0.14, 0.03)	0.201
> 40	0.36 (0.23, 0.49)	< 0.001	–0.09 (–0.18, 0.01)	0.064
Sex (women compared with men)	0.34 (0.20, 0.48)	< 0.001	0.01 (–0.10, 0.10)	0.950
Smoking status^*b*^
Never	Reference		Reference
Past	–0.01 (–0.23, 0.22)	0.939	–0.05 (–0.21, 0.10)	0.529
Current	0.11 (–0.05, 0.26)	0.179	0.08 (–0.03, 0.19)	0.168
Diabetes history	–0.19 (–0.53, 0.14)	0.257	–0.22 (–0.46, 0.01)	0.060
Education length (years)^*b*^
0	Reference		Reference
0–5	0.18 (0.06, 0.29)	0.003	0.06 (–0.02, 0.14)	0.135
> 5	0.28 (0.16, 0.41)	< 0.001	0.02 (–0.07, 0.10)	0.669
BMI baseline (kg/m^2^)^*b*^
≤ 18.09	–0.03 (–0.15, 0.10)	0.660	0.07 (–0.01, 0.16)	0.089
18.09–20.45	Reference		Reference
> 20.45	0.19 (0.06, 0.31)	0.003	–0.07 (–0.16, 0.01)	0.09
Abbreviations: SBP, systolic blood pressure; DBP, diastolic blood pressure; BMI, body mass index.^***a***^When one variable was put in the model, all other variables were adjusted in the same model. ^***b***^Categorized by tertiles.				

Tables 3 and 4 show the associations of arsenic exposure categorized into quartiles and annual change in SBP or DBP. For SBP, we observed a positive association without a dose–response relationship throughout three models; individuals in the higher three quartiles of baseline water arsenic or urinary creatinine-adjusted arsenic had a greater annual increase in SBP compared with those in the reference group (β = 0.43–0.54 mmHg/year and β = 0.39–0.44 mmHg/year for water arsenic and urinary creatinine-adjusted arsenic, respectively) in fully adjusted models (Table 3). Likewise, for DBP, a positive relationship was also observed; individuals in the higher three quartiles of baseline arsenic exposure had a greater annual increase in DBP (β = 0.39–0.41 mmHg/year, and β = 0.37–0.45 mmHg/year for water arsenic and urinary creatinine-adjusted arsenic, respectively) in fully adjusted models compared with those in the lowest quartile (Table 4). For DBP there was a monotonic increase in the rate with increasing urinary creatinine-adjusted arsenic (Table 4). Analyses using different categories of arsenic exposure (tertiles or quintiles) showed similar results (see Supplemental Material, Tables S2 and S3). Sensitivity analyses were conducted by excluding all subjects who were under treatment for hypertension at baseline or follow-up (*n* = 545), without change in the overall results (data not shown). In an analysis of associations with changes in creatinine-adjusted urinary arsenic over time, with the least amount of change between visits (creatinine-adjusted urinary arsenic at later visit minus creatinine-adjusted urinary arsenic at earlier visit) as the reference group (ranging from a decrease of 9 to an increase of 39 μg/g creatinine), the greatest increase (> 39 μg/g creatinine) had a positive but nonsignificant association with the mean annual increase in SBP [β = 0.40; 95% confidence interval (CI): –0.04, 0.83] and DBP (β = 0.28; 95% CI: –0.03, 0.59), whereas there was no association with a decrease of > 9 μg/g creatinine over follow-up (data not shown). Because mean SBP and DBP both were highest at the second follow-up visit (suggesting a possible systematic error in measurement), we repeated analyses excluding follow-up 2 data but found similar results to analyses including data from all visits (data not shown).

**Table 3:** Relation of baseline water arsenic (*N *= 10,853) and baseline urinary creatinine-adjusted arsenic (*N *= 10,549) with adjusted annual changes in systolic blood pressure (SBP) over 7 years of follow-up.

Baseline exposure	Range	Model 1 change/year (mmHg)	Model 2 change/year (mmHg)	Model 3 change/year (mmHg)
Water arsenic (μg/L)
Q1	< 12	Reference	Reference	Reference
Q2	12–62	0.45 (0.32, 0.58)	0.42 (0.29, 0.56)	0.43 (0.29, 0.56)
Q3	62–148	0.60 (0.46, 0.73)	0.55 (0.42, 0.68)	0.54 (0.40, 0.67)
Q4	> 148	0.51 (0.38, 0.65)	0.48 (0.34, 0.61)	0.48 (0.35, 0.61)
Urinary creatinine-adjusted arsenic (μg/g creatinine)
Q1	< 106	Reference	Reference	Reference
Q2	106–199	0.40 (0.26, 0.53)	0.38 (0.25, 0.52)	0.39 (0.25, 0.52)
Q3	199–352	0.45 (0.32, 0.59)	0.43 (0.30, 0.57)	0.44 (0.30, 0.58)
Q4	> 352	0.45 (0.31, 0.58)	0.41 (0.27, 0.54)	0.43 (0.29, 0.56)
Abbreviations: Q1, quartile 1; Q2, quartile 2; Q3, quartile 3; Q4, quartile 4. Model 1: controlled for baseline age and sex. Model 2: controlled for model 1 covariates plus BMI, smoking status, educational status, and history of diabetes. Model 3: controlled for model 2 covariates plus change of urinary creatinine-adjusted arsenic since baseline.				

**Table 4:** Relation of baseline water arsenic (*N *= 10,846), baseline urinary creatinine-adjusted arsenic (*N *= 10,549) with adjusted annual changes in diastolic blood pressure (DBP) over 7 years of follow-up.

Baseline exposure	Range	Model 1 change/year (mmHg)	Model 2 change/year (mmHg)	Model 3 change/year (mmHg)
Water arsenic (μg/L)
Q1	< 12	Reference	Reference	Reference
Q2	12–62	0.44 (0.35, 0.53)	0.42 (0.33, 0.52)	0.41 (0.31, 0.50)
Q3	62–148	0.47 (0.38, 0.56)	0.42 (0.33, 0.52)	0.41 (0.32, 0.51)
Q4	> 148	0.43 (0.34, 0.52)	0.40 (0.31, 0.49)	0.39 (0.30, 0.49)
Urinary creatinine-adjusted arsenic (μg/g creatinine)
Q1	< 106	Reference	Reference	Reference
Q2	106–199	0.38 (0.29, 0.48)	0.37 (0.27, 0.46)	0.37 (0.27, 0.46)
Q3	199–352	0.40 (0.30, 0.49)	0.37 (0.27, 0.46)	0.38 (0.28, 0.47)
Q4	> 352	0.49 (0.40, 0.58)	0.45 (0.35, 0.54)	0.45 (0.36, 0.55)
Abbreviations: Q1, quartile 1; Q2, quartile 2; Q3, quartile 3; Q4, quartile 4. Model 1: controlled for baseline age and sex. Model 2: controlled for model 1 covariates plus BMI, smoking status, educational status, and history of diabetes. Model 3: controlled for model 2 covariates plus change of urinary creatinine-adjusted arsenic since baseline.				

Last, we assessed the association between baseline arsenic exposure and the absolute levels of BP at the third follow-up (see Supplemental Material, Figure S1). In fully adjusted models, individuals with the highest level of baseline water arsenic had 3.95 mmHg (95% CI: 3.15, 4.76) greater SBP or 2.65 mmHg (95% CI: 2.21, 3.31) greater DBP compared with those in the reference group. Similarly, for urinary creatinine-adjusted arsenic, individuals with higher concentrations had a 3.47 mmHg (95% CI: 2.61, 4.33) increase in SBP or a 2.62 mmHg (95% CI: 1.95, 3.03) increase in DBP compared with those in the lowest quartile. However, associations were similar across quartiles 2, 3, and 4, without evidence of a monotonic trend (see Supplemental Material, Figure S1).

## Discussion

To our knowledge, the present study is the first large epidemiologic study to examine the relationship between arsenic exposure from drinking water and longitudinal change in BP. We found positive associations of arsenic exposure, measured either in well water or urine samples, with annual change in SBP and DBP, over an average of 6.7 years of follow-up.

The association of arsenic exposure with BP has been indicated in several cross-sectional studies. A systematic review including 11 cross-sectional studies reported a pooled OR of 1.27 (95% CI: 1.09, 1.47; *p*-value for heterogeneity = 0.001) for high BP comparing the highest and lowest arsenic exposure categories (Abhyankar et al. 2012). However, cross-sectional assessments of the association between arsenic exposure and BP are limited by *a*) possible selection bias in capturing only individuals who have lived long enough, and *b*) limited detection of the latent effects of arsenic exposure on BP. In contrast, longitudinal analyses mitigate some of these problems and may be a superior method for examining arsenic exposure on BP change over time. Longitudinal analyses have previously revealed the effects of lead exposure on BP change (Glenn et al. 2003, 2006). Our findings demonstrating an association between arsenic exposure and annual BP change contributes to the growing body of evidence indicating that environmental exposures may play a role in longitudinal BP change.

We did not find a monotonic relationship between arsenic exposure and the slope of BP change over time. In our previous cross-sectional study, the positive association between arsenic exposure from drinking water and baseline BP also was not stronger with increasing quartiles of arsenic exposure (Chen Y et al. 2007a). Mechanistic studies have indicated that the vascular effect of arsenic may be nonlinear (Soucy et al. 2003) and may reach threshold when arsenic exposure exceeds a certain level. Alternatively, the baseline BP may have been affected by arsenic exposure already leading to a limited increase on the rate of BP change that can be further observed. In addition, the increased rate of BP change may be limited in this relatively young cohort.

In the present study, exposure to water with arsenic concentrations > 12 μg/L was associated with a greater increase of 0.43–0.54 mmHg/year and 0.39–0.41 mmHg/year for SBP and DBP, respectively. Evidence suggests that the risk of CVD rises continuously as both SBP and DBP increase from 115 mmHg and 75 mmHg, respectively (Lewington et al. 2002). Based on estimates from 61 prospective observational studies, even a 2-mmHg decrease in usual SBP would involve about 10% lower stroke mortality and about 7% lower mortality from ischemic heart disease or other vascular causes in middle age (Lewington et al. 2002). Although the estimate may not be the same in our study population, given the strong association between BP and CVD risk, the differences in the rate of BP change associated with arsenic exposure, although small in magnitude annually, may have a cumulative effect on the risk of clinical events.

The potential association between arsenic exposure and high BP is supported by experimental studies. *In vitro* work has shown that arsenic promotes inflammatory activity, oxidative stress, and endothelial dysfunction through several mechanisms, including the activation of stress-response transcription factors such as activator protein-1 and nuclear factor-κB (Abhyankar et al. 2012). In animal models, chronic exposure of rats and rabbits to arsenite has been shown to cause a considerable increase in peripheral vascular resistance (Abir et al. 2012). In rats, lifelong arsenic exposure increased BP after only 80 days, and elevations persisted through 200 days (Yang et al. 2007). Furthermore, arsenic exposure may also be related to renal dysfunction, leading to BP changes in individuals (Chen JW et al. 2011; Hsueh et al. 2009).

Our study, which is among the first to prospectively investigate the role of arsenic exposure in longitudinal BP change, has several strengths. First, we have obtained multiple research-quality BP measurements over 7 years of follow-up, which enables us to depict BP longitudinal change over time. Second, the low percentage of the population using antihypertensive medications (around 1% at baseline) and the absence of alcohol consumption due to religious beliefs allowed us to investigate BP change without the influence of medical therapy or alcohol. Finally, we have a rich set of covariates that allow us to adjust for confounders.

The study also has limitations. Although we used the same methodology for measuring BP since baseline and for follow-ups, measurement errors for BP measurements could have occurred. We could not estimate the extent of the potential measurement errors. However, the relationships between conventional risk factors and longitudinal change in BP were consistent with those of the literature, supporting the validity of the BP measurement in this study. Also, sensitivity analysis excluding follow-up 2, which was conducted out of concern for error at follow-up 2, generated similar results. The self-reported well water use might also produce misclassifications of exposure. However, the correlation of well water arsenic concentration and urinary creatinine-adjusted arsenic at baseline was high (ρ = 0.70), supporting the validity of self-reported data on well use and population-wide arsenic exposure in this population. The analyses were restricted to individuals with available data on repeated BP measurements. Because arsenic exposure has been related to CVD mortality in the cohort and high BP is a CVD risk factor, the exclusion of individuals without repeated BP measurements may have preferentially removed individuals with high BP that is associated with arsenic, leading to a potential bias toward the null on average. However, missing BP was not extensive (9,069 subjects have complete BP measurements), and the demographic distributions of our study population and the overall population were very similar (see Supplemental Material, Table S1). In the analyses, we controlled for visit-to-visit changes in urinary creatinine-adjusted arsenic. However, given that arsenic exposure was similar in the majority of participants, we may not have power to assess the effects of changes in urinary creatinine-adjusted arsenic. We did not assess the role of specific nutrients or nutritional intake in the present study. Future studies are needed to investigate whether the association of arsenic exposure and the rate of BP change differs by nutritional status.

## Conclusion

We found positive associations between long-term arsenic exposure and BP increase over time, which might be one mechanism by which arsenic may lead to CVD. Further studies are needed to investigate other preclinical indicators or biomarkers of CVD with multiple measurements.

## Supplemental Material

(391 KB) PDF
