# Supplementary material for: Association between Arsenic Exposure from Drinking Water and Longitudinal Change in Blood Pressure among HEALS Cohort Participants
Source: Environ Health Perspect. 2015 Mar 27;123(8):806–12. doi: 10.1289/ehp.1409004 (PMC4529016; doi:10.1289/ehp.1409004)
Supplement: (391 KB) PDF [file ehp.1409004.s001.acco.pdf]

Environ Health Perspect
doi: 10.1289/ehp.1409004

Note to Readers: ***EHP*** **strives to ensure that all journal content is accessible to all readers. However, some figures and Supplemental Material published in *EHP* articles may not conform to 508 standards due to the complexity of the information being presented. If you need assistance accessing journal content, please contact ehp508@niehs.nih.gov. Our staff will work with you to assess and meet your accessibility needs within 3 working days.**

Supplemental Material

Association between Arsenic Exposure from Drinking Water and Longitudinal Change in Blood Pressure among HEALS Cohort Participants

Jieying Jiang, Mengling Liu, Faruque Parvez, Binhuan Wang, Fen Wu, Mahbub Eunus, Sripal Bangalore, Jonathan D. Newman, Alauddin Ahmed, Tariqul Islam, Muhammad Rakibuz-Zaman, Rabiul Hasan, Golam Sarwar, Diane Levy, Vesna Slavkovich, Maria Argos, Molly Scannell Bryan, Shohreh F. Farzan, Richard B. Hayes, Joseph H. Graziano, Habibul Ahsan, and Yu Chen

Table of Contents

Details on arsenic exposure in the population

References

Table S1. Demographic, lifestyle factors and arsenic exposure variables in the overall and study populations.

Table S2. Relation of baseline water arsenic, baseline urinary creatinine-adjusted arsenic and adjusted annual changes in BP within 7 years, by tertiles of arsenic exposure.

Table S3. Relation of baseline water arsenic, baseline urinary creatinine-adjusted arsenic and adjusted annual changes in BP within 7 years, by quintiles of arsenic exposure.

Figure S1. Adjusted means of blood pressure at the third visit by quartiles of baseline arsenic in water or urine. * P<.001 when compared with reference group (Q1). Model was adjusted for baseline age, sex, baseline BMI, baseline smoking status, baseline educational status and history of diabetes.

Details on arsenic exposure in the population

Wells shared by one to six participants comprised 86% of the overall HEALS participants (N = 10,494), and no more than 14 individuals were recruited from any given well (Ahsan et al. 2006). Well arsenic was the main exposure in the population. Well-water arsenic was correlated with total urinary arsenic, urinary dimethylarsinic acid (DMA), and urinary monomethylarsonic acid (MMA) concentration in our study population (of 0.70, 0.61, and 0.57, respectively) and therefore was clearly the primary source of arsenic in the urine (Chen et al. 2010). While inorganic forms of arsenic found in rice may also contribute total arsenic exposure measured by urinary arsenic, the correlation between rice consumption and total urinary arsenic was weak in our population (ρ =0.03) (Chen et al. 2010).

Arsenic exposure level remained similar in the majority of the population. The average level of urinary creatinine-adjusted arsenic was 285 µg/g at baseline, 224 µg/g at follow-up 1, 220 µg/g at follow-up 2, and 210 µg/g at follow-up 3. Overall, total urinary creatinine-adjusted arsenic decreased by an average of 61 µg/g from baseline to first follow-up and then essentially remained stable with an average decrease of 7 µg/g from first to third follow-up. The correlation of baseline urinary creatinine-adjusted arsenic with urinary arsenic measured at each of the three follow-up visits were all around 0.60.

References

Ahsan H, Chen Y, Parvez F, Argos M, Hussain AI, Momotaj H, et al. 2006. Health effects of arsenic longitudinal study (heals): Description of a multidisciplinary epidemiologic investigation. J Expo Sci Environ Epidemiol 16:191-205.

Chen Y, Ahsan H, Slavkovich V, Peltier GL, Gluskin RT, Parvez F, et al. 2010. No association between arsenic exposure from drinking water and diabetes mellitus: A cross-sectional study in bangladesh. Environ Health Perspect 118:1299-1305.

Table S1. Demographic, lifestyle factors and arsenic exposure variables in the overall and study populations.

Characteristics	Overall population		Study population	
	No.	Mean ± SD or %	No.	Mean ± SD or %
**Age, years**	11746	37.1 ± 10.1	10853	36.8 ± 10.0
**Male , %**	5042	42.9	4588	42.3
**Ever smoker , %**	4172	35.5	3788	34.9
**Diabetes history , %**	241	2.1	203	1.9
**Education, years**	11740	3.5 ± 3.8	10853	3.4 ± 3.8
**BMI baseline, kg/m^2**	11467	19.8 ± 3.2	10791	19.7 ± 3.1
**Systolic Blood Pressure, mmHg**				
Baseline	11487	114.7 ± 17.9	10853	113.8 ± 16.8
Follow up 1	11130	114.6 ± 17.9	10577	113.8 ± 17.1
Follow up 2	10753	119.0 ± 16.8	10013	117.9 ± 15.4
Follow up 3	10590	113.0 ± 16.7	9553	111.4 ± 15.4
**Diastolic Blood Pressure, mmHg**				
Baseline	11481	74.0 ± 11.8	10846	73.4 ± 11.3
Follow up 1	11130	73.3 ± 10.6	10577	72.9 ± 10.2
Follow up 2	10754	76.8 ± 10.5	10013	76.2 ± 10.0
Follow up 3	10590	73.9 ± 10.6	9553	73.1 ± 10.1
**Water arsenic, µg/L**				
Baseline	11746	101.5 ± 115.4	10853	101.7 ± 115.7
Follow up 1	9405	62.8 ± 91.4	8940	63.2 ± 91.9
Follow up 2	8816	52.6 ± 80.9	8386	53.2 ± 81.6
Follow up 3	7140	50.7 ± 78.4	6798	51.1 ± 79.1
**Urinary arsenic, µg/L**				
Baseline	11224	137.7 ± 156.9	10549	138.7 ± 158.4
Follow up 1	11109	122.1 ± 137.9	10558	122.8 ± 139.4
Follow up 2	10762	118.7 ± 132.8	10244	119.0 ± 133.0
Follow up 3	10562	102.7 ± 116.8	10057	102.6 ± 116.9

Table S2. Relation of baseline water arsenic, baseline urinary creatinine-adjusted arsenic and adjusted annual changes in BP within 7 years, by tertiles of arsenic exposure.

**Baseline water arsenic (μg/L) N=10,853**	**T1 <25**	**T2 25-114**	**T3 >114**
**SBP**	Change/year (mmHg)	Change/year (mmHg)	Change/year (mmHg)
Model 3[a]	Ref.	0.49 (0.37,0.60)	0.45 (0.33,0.57)
**DBP**			
Model 3[a]	Ref.	0.38 (0.29,0.46)	0.31 (0.22,0.39)
**Baseline urinary creatinine-adjusted arsenic (μg/g creatinine) N=10,549**	**T1 <134**	**T2 134-285**	**T3 >285**
**SBP**	Change/year (mmHg)	Change/year (mmHg)	Change/year (mmHg)
Model 3[a]	Ref.	0.32 (0.20,0.44)	0.36 (0.24,0.48)
**DBP**			
Model 3[a]	Ref.	0.29 (0.21,0.38)	0.37 (0.28,0.45)

Abbreviations: BP, blood pressure; SBP: systolic blood pressure, DBP: diastolic blood pressure, T1, tertile 1; T2, tertile 2; T3, tertile 3

[a]Controlled for baseline age, sex, BMI, smoking status, educational status, history of diabetes and change of urinary creatinine-adjusted arsenic since baseline.

Table S3. Relation of baseline water arsenic, baseline urinary creatinine-adjusted arsenic and adjusted annual changes in BP within 7 years, by quintiles of arsenic exposure.

**Baseline water arsenic (μg/L) N=10,853**	**Q1 <7**	**Q2 7-39**	**Q3 39-91**	**Q4 91-179**	**Q5 >179**
**SBP**	Change/year (mmHg)	Change/year (mmHg)	Change/year (mmHg)	Change/year (mmHg)	Change/year (mmHg)
Model 3[a]	Ref.	0.32 (0.17,0.48)	0.56 (0.41,0.72)	0.55 (0.40,0.70)	0.48 (0.33,0.63)
**DBP**					
Model 3[a]	Ref.	0.31 (0.21,0.42)	0.44 (0.33,0.54)	0.36 (0.26,0.47)	0.45 (0.35,0.55)
**Baseline urinary creatinine-adjusted arsenic (μg/g creatinine) N=10,549**	**Q1 <91**	**Q2 91-160**	**Q3 160-248**	**Q4 248-409**	**Q5 >409**
**SBP**	Change/year (mmHg)	Change/year (mmHg)	Change/year (mmHg)	Change/year (mmHg)	Change/year (mmHg)
Model 3[a]	Ref.	0.25 (0.10,0.40)	0.37 (0.22,0.53)	0.45 (0.30,0.60)	0.41 (0.26,0.56)
**DBP**					
Model 3[a]	Ref.	0.31 (0.20,0.41)	0.39 (0.28,0.50)	0.42 (0.32,0.53)	0.49 (0.39,0.60)

Abbreviations: BP, blood pressure; SBP: systolic blood pressure, DBP: diastolic blood pressure, Q1, quintile 1; Q2, quintile 2; Q3, quintile 3; Q4 quintile 4; Q5 quintile 5.

[a]Controlled for baseline age, sex, BMI, smoking status, educational status, history of diabetes and change of urinary creatinine-adjusted arsenic since baseline.

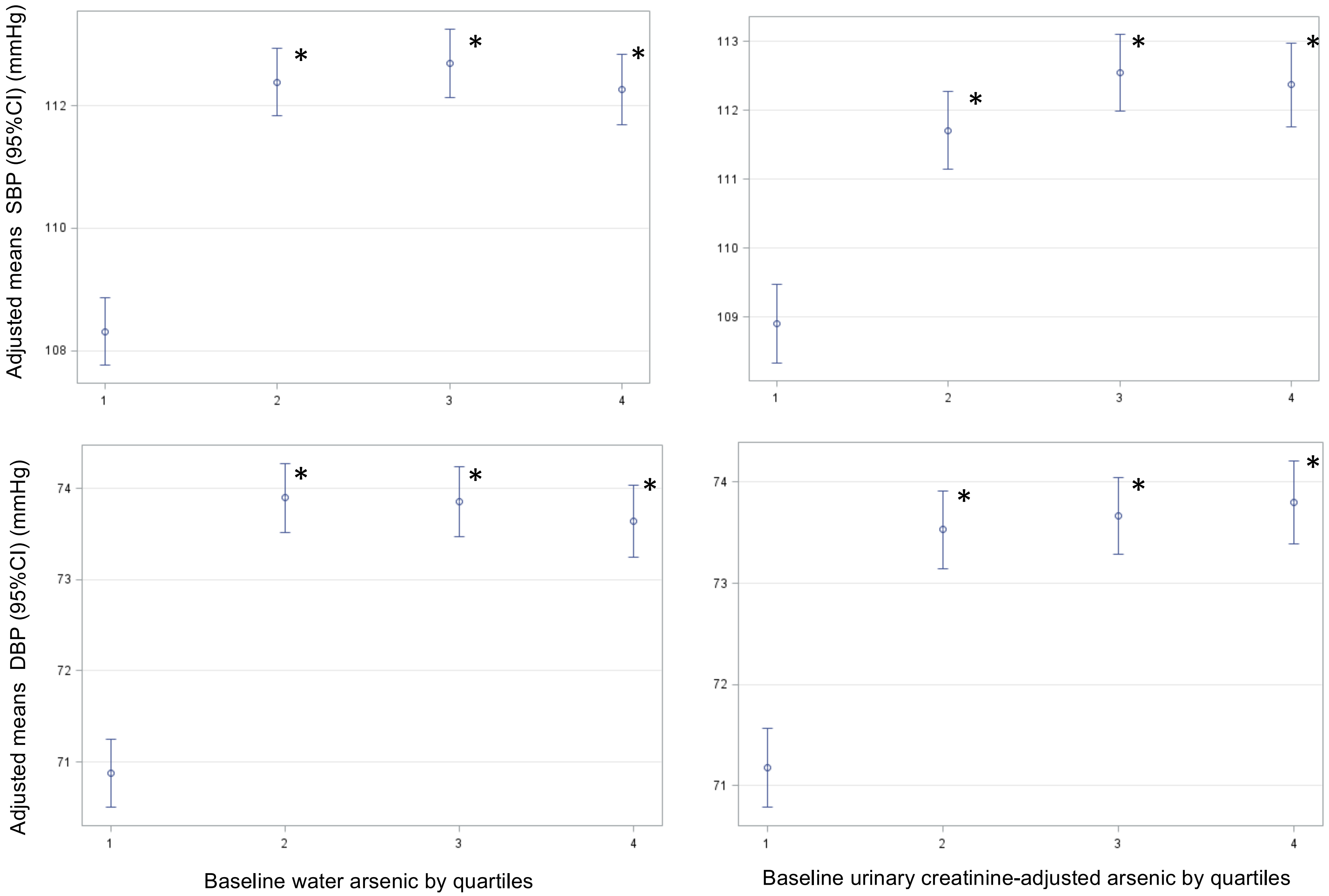

Figure S1. Adjusted means of blood pressure at the third visit by quartiles of baseline arsenic in water or urine. * P<.001 when compared with reference group (Q1). Model was adjusted for baseline age, sex, baseline BMI, baseline smoking status, baseline educational status and history of diabetes.
